# Enhancing Carbon Nanotube Yarns via Infiltration Filling with Polyacrylonitrile in Supercritical Carbon Dioxide

**DOI:** 10.3390/molecules29143404

**Published:** 2024-07-20

**Authors:** Baihua Liu, Zhifeng Hu, Zeyu Sun, Muhuo Yu

**Affiliations:** 1 State Key Laboratory for Modification of Chemical Fibers and Polymer Materials, College of Materials Science and Engineering, Donghua University, Shanghai 201620, China; bhliu@mail.dhu.edu.cn (B.L.);; 2 Center for Civil Aviation Composites, Shanghai Key Laboratory of Lightweight Composites, Donghua University, Shanghai 201620, China

**Keywords:** carbon nanotube yarns, swelling and high permeability effect, supercritical carbon dioxide

## Abstract

Carbon nanotube (CNT) fibers are renowned for their exceptional axial tensile strength and modulus. However, in yarn form, they frequently encounter transverse loading in practical applications, which exposes their suboptimal mechanical attributes rooted in inadequate inter-tube interactions and yarn surface defects. Efforts to mitigate micro-slippage among CNTs have encompassed gap-filling methodologies with varied materials, yet the outcomes have fallen short of expectations. This work aimed to enhance the mechanical properties of CNT yarns via infiltration with polyacrylonitrile (PAN) under supercritical carbon dioxide (sc-CO_2_) conditions. PAN was strategically chosen for its capability to undergo pre-oxidation and subsequent carbonization, leading to robust graphitic reinforcement. Leveraging sc-CO_2_’s swelling and high permeability properties, the infiltration process effectively plugged interstitial spaces, elevating the yarn’s tensile strength to 277.50 MPa and Young’s modulus to 5094.05 MPa. Additional enhancements were realized after pre-oxidation, conferring a dense, reinforced shell structure that augmented tensile strength by 96.93% and Young’s modulus by 298.80%. Scanning electron microscopy (SEM) analyses revealed a homogeneous PAN distribution within the yarn matrix, corroborated by X-ray photoelectron spectroscopy (XPS) evidence of C-N bonding, indicative of a successfully interlaced network. Consequently, this investigation introduces a novel strategy to tackle micro-slippage in CNT yarns, thereby achieving substantial improvements in their mechanical resilience.

## 1. Introduction

Carbon nanotubes (CNTs) have attracted extensive research attention due to their outstanding mechanical properties and electrical conductivity [1,2,3]. As macroscopic assemblies, CNT yarns hold promise for expanded engineering applications [4,5,6]. However, their mechanical performance falls short of aspirations, primarily due to insufficient lateral interactions among CNTs. This deficiency stems from the inherently smooth and low-friction surfaces of CNTs, which is crucial to optimizing the mechanical performance of CNT yarns. The modification of carbon nanotubes can enhance the interaction of carbon nanotubes with the polymer matrix [7]. Nevertheless, for the CNT yarns obtained through the array spinning method, it is extremely difficult to commence the modification from the origin of the carbon nanotube array. Polyacrylonitrile (PAN), a prospective polymeric additive, holds the potential to enhance the properties of the matrix through the formation of robust C-C bonds [8,9]. By leveraging the swelling capacity and high permeability of supercritical carbon dioxide (sc-CO_2_), PAN infiltrates the as-spun CNT yarn matrix, effectively occupying inter-bundle voids and facilitating C-C bond creation [10]. Subsequently, a pre-oxidation treatment is performed to form a ring structure within the yarn, further enhancing its mechanical properties.

Scholars worldwide currently concentrate on investigating the fabrication methodologies of CNT yarns, including solution spinning [11,12], array spinning [13,14,15,16,17,18,19], and floating chemical vapor deposition spinning [5]. In the past two decades, researchers have dedicated efforts to developing continuous spinning processes for CNT yarns and have delved deeply into understanding the relationships between the spinning processes and the structural performance of the yarns. These render CNT yarns highly appealing for applications within aerospace, aviation, and automotive manufacturing, where their superior mechanical properties are crucial [20]. Various modification strategies for CNT yarns have been explored, including fiber densification using solvent shrinkage, twisting [21], polymer infiltration with polymers like polyvinyl alcohol (PVA), polyimide (PI), and polyacrylonitrile (PAN) [22,23,24], surface modification utilizing azo compounds [21], thermal treatment, and exposure to high-temperature radiation or welding processes [25].

**Figure 1:**
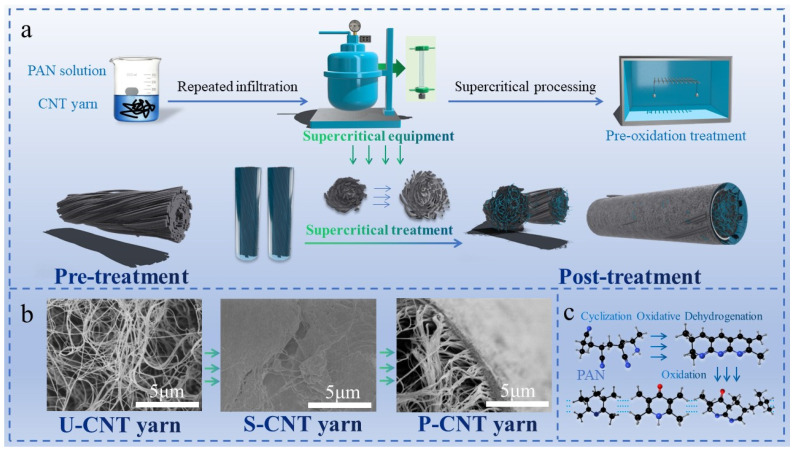
(**a**) Process and mechanism diagram of CNT yarn treatment; (**b**) SEM images of cross-section of CNT yarn before and after treatment; (**c**) chemical changes in PAN during pre-oxidation process.

Supercritical fluids, existing beyond their critical temperature and pressure thresholds, exhibit a hybrid of liquid and gaseous properties. Characterized by high solubility, density, viscosity, a high diffusion coefficient, and solvation ability, these fluids are pivotal in various applications [26,27]. Notably, common choices include water, ethylene, ammonia, methanol, ethanol, and supercritical carbon dioxide (sc-CO_2_) [28,29,30]. The sc-CO_2_ combines a near-liquid density and viscosity with a gas-like diffusion coefficient, which is approximately 100 times greater than typical liquids, thereby significantly enhancing its solvating capability. It varies with temperature and pressure, which can be manipulated to optimize the swelling of polymers and the distribution of penetrating agents, thereby improving the adsorption and dissolution effects on polymer monomers and enhancing the diffusion rates of these agents. By leveraging the plasticizing and swelling effects of supercritical CO_2_, polyacrylonitrile solution is introduced into the interior of CNT yarns to address internal defects and mitigate micro-slip issues. Following this, a pre-oxidation process cyclizes the polyacrylonitrile, forming chemical bonds that not only fill internal gaps but also create connections between carbon nanotubes, reducing slip. This process substantially improves the mechanical properties of the yarns, and the processing process and mechanism diagram are shown in Figure 1. We also explored the optimal concentrations of polyacrylonitrile solution and the best experimental conditions for its application using supercritical CO_2_. An orthogonal experimental method was employed to determine the optimal conditions for the actions of supercritical CO_2_ and pre-oxidation processes.

Compared with previous carbon deposition or infiltration of other polymers, this paper pioneered the use of the supercritical carbon dioxide treatment method to introduce polyacrylonitrile into the interior of CNT yarns to repair the micro-slip problem between CNT yarns. In this study, we introduced PAN molecules into the interior of CNT yarns through the swelling and permeation of supercritical carbon dioxide for the first time. By observing the apparent properties and characterizing the mechanical properties of CNT yarns with varying infiltration times, the optimal infiltration times are selected. The optimal conditions for supercritical carbon dioxide treatment, including pressure, temperature, and time, were analyzed using orthogonal experiments. Further pre-oxidation treatment was performed on the S-CNT yarn, and the optimal pre-oxidation conditions, including tension, temperature, and time period, were analyzed using orthogonal experiments. By comparing the morphology, mechanical properties, and elemental analysis of carbon nanotube yarns at different stages, it was confirmed that PAN effectively penetrated the interior of carbon nanotube yarns and formed a unique shell structure, thereby improving the mechanical properties.

## 2. Results and Discussions

CNT yarns hold significant promise as emerging high-performance carbon nanomaterials. Leveraging the swelling and permeability properties of supercritical carbon dioxide (scCO_2_) enables the effective infiltration of polyacrylonitrile (PAN) into the CNT yarn structure, thereby tackling the prevalent issue of substantial micro-slippage. [31].

### 2.1. The Effect of Polyacrylonitrile Solution Concentration and Infiltration Times on the CNT Yarn

**Figure 2:**
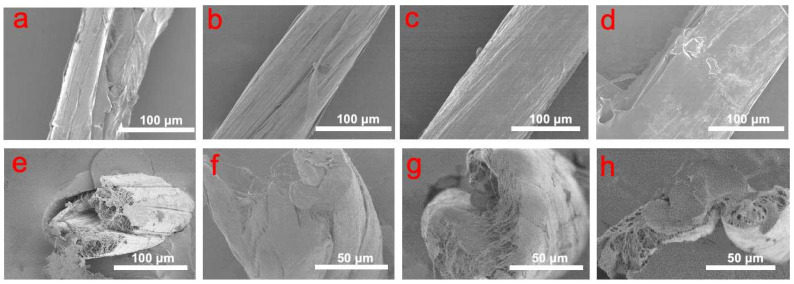
SEM images of the surfaces and cross-section of CNT yarns after infiltration with different concentrations of polyacrylonitrile solution. (**a**,**e**) 0 C* (**b**,**f**) 1/3 C* (**c**,**g**) 1/2 C* (**d**,**h**) C*.

Figure 2 presents surface SEM images elucidating the effect of varied PAN concentrations on the CNT yarns’ morphology. Specifically, images (a) through (d), labeled as 0 C*, 1/3 C*, 1/2 C*, and C*, respectively, depict an ascending trend in surface deposition with increased PAN concentration. At 1/2 C* concentration, a significantly thin PAN layer is visible, effectively compensating for surface defects. However, at C* concentration, the excessive deposition may affect its mechanical properties. Cross-sectional SEM images (Figure 2e–h) provide complementary evidence, showing that untreated yarns have numerous voids, which limit their mechanical properties. These images correspond to concentrations 0 C*, 1/3 C*, 1/2 C*, and C*, respectively. The analysis suggests that 1/2 C* constitutes the optimal infiltration concentration, achieving an equilibrium between superficial coverage and penetration depth. This concentration adequately remedies both superficial defects and internal voids without causing yarn saturation.

**Figure 3:**
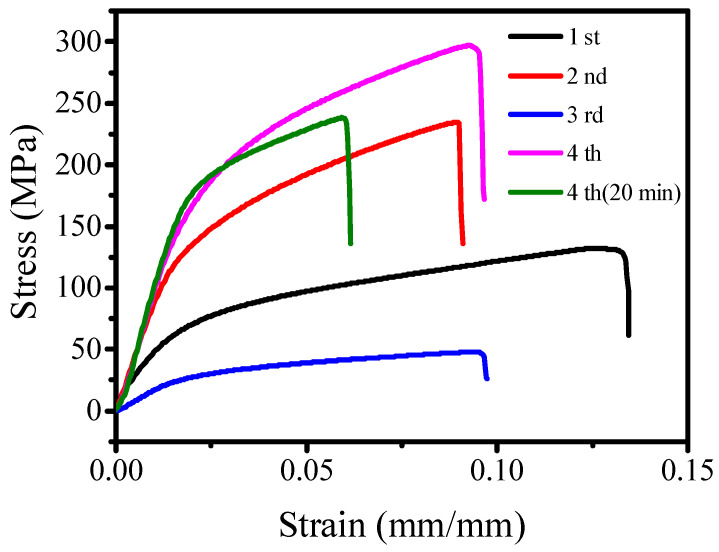
Strain–stress curve of CNT yarn modified by supercritical CO_2_ with repeated infiltration.

To ensure complete infiltration of CNT yarn, the influence of different infiltration times was studied. A repeated infiltration method was employed, with the yarn being infiltrated from 1 to 4 times for a duration of 10 min. Following each infiltration, the CNT yarns were treated with a one-hour exposure to sc-CO_2_. To verify the relationship between infiltration duration and the mechanical characteristics of CNT yarns, the infiltration time was extended to 20 min per cycle. Figure 3 illustrates the mechanical performance of CNT yarns after varied infiltration durations and times with supercritical CO_2_ treatments. The data indicate a substantial improvement in mechanical properties with increased infiltration times. Specifically, the maximum mechanical strength of 298 MPa was achieved following the fourth infiltration cycle (noted as 4th). However, extending the duration of the sc-CO_2_ treatment to 20 min, the mechanical strength decreased, although the modulus increased. Consequently, the optimized infiltration conditions were established as four cycles of 10-min infiltrations with one-hour sc-CO_2_ treatment every time.

### 2.2. Determination of the Optimal Process Conditions for Modifying CNT Yarn with Supercritical CO_2_ Fluid

To identify the optimal treatment conditions, an orthogonal experimental method was employed, configuring three levels and three factors, as detailed in Table 1. The specific parameters adjusted were pressure (8 MPa, 10 MPa, 12 MPa), temperature (100 °C, 150 °C, 200 °C), and tension (1.23 cN, 4.06 cN, 8.40 cN).

**Figure 4:**
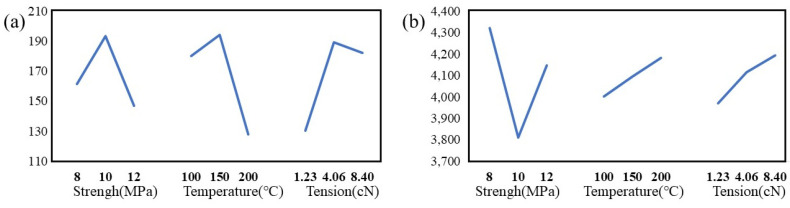
Effect curve of strength (**a**) and modulus (**b**) factors on CNT yarns treated with sc-CO_2_.

The mechanical properties of CNT yarns post-treatment were analyzed via an Instron tensile testing machine. The results of tensile strength and tensile modulus are shown in Table 2, and the strength and modulus factor effect curve are displayed in Figure 4 These illustrate the impact of varying sc-CO_2_ processing conditions on the yarns’ mechanical properties.

The orthogonal experiment yielded an optimal configuration denoted as A2, B2, and C3, corresponding to conditions of 10 MPa pressure, 150 °C temperature, and 8.40 cN tension. Under these conditions, the strength of the S-CNT yarn reaches 277.50 MPa, which is 109.78% higher than the strength of untreated CNT yarn (U-CNT yarn) of 132.28 MPa. This result indicates that the temperature has the most significant impact on yarn strength, followed by tension and pressure. Specifically, the temperature variation affects how the PAN molecules swell and permeate into the yarns, enhancing the yarn’s structural integrity. An optimal experimental configuration of A3 B3 C2 was determined, achieving a modulus of 5437.84 MPa, representing a significant 114.28% increase over the modulus of 2537.74 MPa observed in the U-CNT yarn. Here, pressure demonstrated the most significant influence on the modulus, indicating that excessively high pressures might overly compress the yarn’s pore structure, thereby impeding PAN infiltration and reducing modulus. Consequently, the optimal conditions for subsequent treatments to maximize modulus were identified as a temperature of 200 °C, a tension of 8.40 cN, and a pressure of 8 MPa.

**Figure 5:**
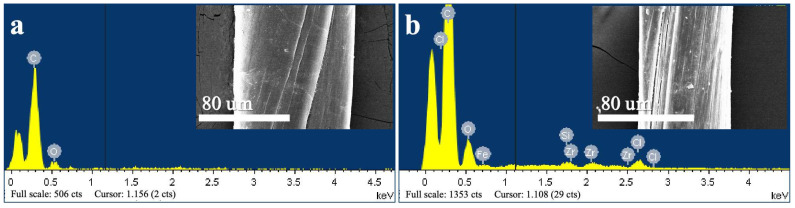
EDS plots of C-CNT yarn (**a**) and S-CNT yarn (**b**).

Further analysis of the C-CNT yarn was conducted utilizing energy dispersive spectroscopy (EDS) to investigate the elemental variations after sc-CO_2_ treatment. As illustrated in Figure 5 and Table 3, a minor reduction in carbon content and a corresponding increase in oxygen content were observed in the S-CNT yarn. This change suggests a degree of carbon nanotube degradation at high temperatures. After supercritical treatment, some metallic elements appeared, potentially due to the interaction with metal elements introduced during the tension application process with metal tools. This incidental metal contamination highlights the importance of considering the materials and conditions used in treatment processes.

### 2.3. Determination of the Optimal Process Conditions for Pre-Oxidation Treated CNT Yarn

During pre-oxidation, PAN polymer chains undergo oxidation reactions, resulting in substantial alterations to their molecular structure, including cross-linking, where individual polymer chains bond chemically to form a more stable network. The PAN molecules introduced into CNT yarns develop a ring structure during pre-oxidation, thereby enhancing the yarns’ strength and modulus. To determine the optimal pre-oxidation conditions for S-CNT yarns, we employed an orthogonal experimental design with three levels and three factors.

The experimental parameters were adjusted as follows:

Temperature Profiles: Three schemes were utilized: (i) five increments (180 °C→200 °C→230 °C→250 °C→280 °C), (ii) seven increments (180 °C→190 °C→200 °C→220 °C→240 °C→260 °C→280 °C), and (iii) ten incremental steps (180 °C to 280 °C in 10 °C steps).

Tension Settings: Applied tensions were set at 1.23 cN, 4.06 cN, 8.40 cN.

Residence Time: Duration varied across three options: 1 h, 1.5 h, 2 h.

**Figure 6:**
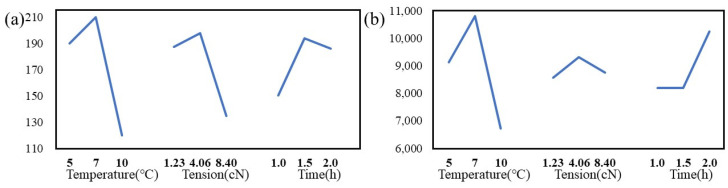
Effect curve of factors affecting the strength (**a**) and modulus (**b**) of CNT yarns treated with pre-oxidation treatment.

Results from the orthogonal experiments, as presented in Table 4, revealed that the optimal conditions for maximizing the strength of P-CNT yarns were achieved using a seven-stage temperature setting, with a tension of 1.23 cN maintained for 1.5 h. And, the strength and modulus factor effect curve are displayed in Figure 6.These conditions yielded a strength of 260.57 MPa, which was slightly inferior to that of S-CNT yarns. Analysis revealed that temperature exerted the most significant influence on yarns’ strength, particularly at lower temperatures where PAN molecules initiate reactions gradually, leading to fundamental changes in the polymer chain. At higher temperature ranges (220–260 °C), the oxidation reaction intensifies, accelerating the cross-linking and stabilization of polymer chains, causing PAN to transition from a light white or yellow to brown. At around 280 °C, the reactions are nearly complete. However, prolonged exposure to high temperatures can compromise the yarn strength. In contrast, for modulus, the optimal conditions entailed a seven-stage temperature setting, coupled with a tension of 4.06 cN maintained for 2 h, resulting in significant enhancement of the modulus to 12,984.42 MPa.

### 2.4. Effects of Pre-Oxidation Treatment and Supercritical CO_2_ Fluid Modification on the Structure and Properties of CNT Yarns

**Figure 7:**
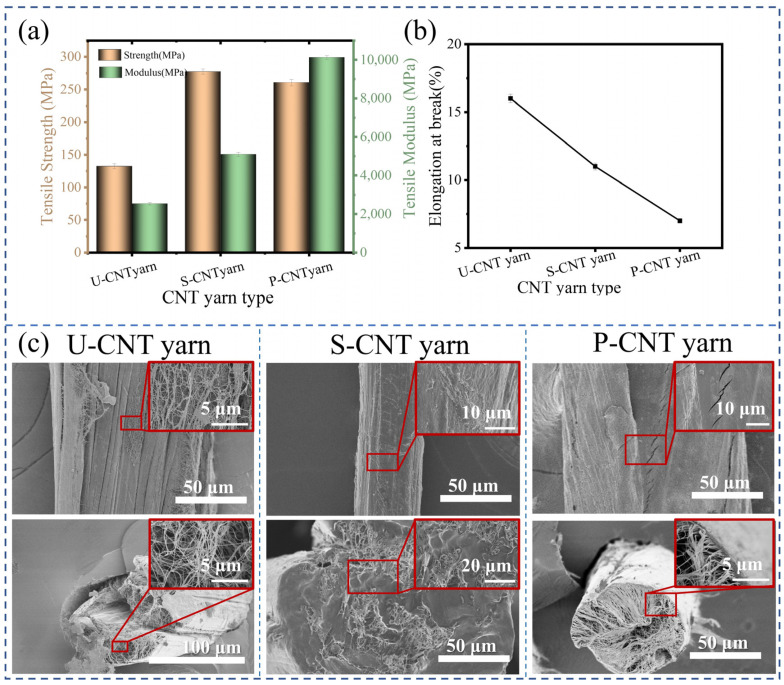
Tensile mechanical properties (**a**), elongation at break (**b**), and SEM images of surface and cross-section (**c**) of CNT yarn.

The mechanical properties of U-CNT yarn, S-CNT yarn, and P-CNT yarn were evaluated under optimal conditions for supercritical CO_2_ fluid modification and pre-oxidation treatment. Figure 7a,b compare the structural performance differences among these samples, specifically the tensile mechanical properties and elongation at the break of CNT yarns at various stages. The figure reveals that the strength of the S-CNT yarn is 277.50 MPa, representing a 110.10% increase compared to the 132.28 MPa strength of the U-CNT yarn. Furthermore, the modulus of the S-CNT yarn increased significantly from 2537.74 MPa to 5094.05 MPa, indicating a 100.73% improvement. Moreover, the P-CNT yarn exhibits a strength of 260.50 MPa, a 96.93% increase over the U-CNT yarn, and a modulus of 10,122.13 MPa, representing a remarkable 298.8% enhancement. From Figure 7b, it is evident that the fracture elongation of CNT yarns significantly decreases after pre-oxidation treatment. After pre-oxidation, the fracture elongation of the U-CNT yarn decreases from 16.12% to 7.03%.

As depicted in Figure 7c, it is apparent that the surface of the U-CNT yarn exhibits a number of defects characterized by significant gaps between the internal carbon nanotubes. In contrast, the surface of S-CNT yarn forms a dense layer of PAN, resulting in a smooth and burr-free surface. The gaps are filled by the internal PAN molecules, leading to a significant improvement in the CNT yarns’ internal defects. This treatment effectively addresses the issue of micro-slip among the carbon nanotubes, thereby approximating the CNT yarn’s strength modulus to the desired ideal value. While the dense surface layer of P-CNT yarn remains intact, its internal structure forms a distinct shell. This phenomenon is attributed to the contraction of PAN molecules during the cyclization process. Inside the CNT yarn, the PAN molecules struggle to withstand tensile forces, causing them to rearrange and aggregate on the inner surface, ultimately resulting in a dense shell structure during thermal cyclization [32]. This dense shell structure significantly enhances the modulus of CNT yarn.

**Figure 8:**
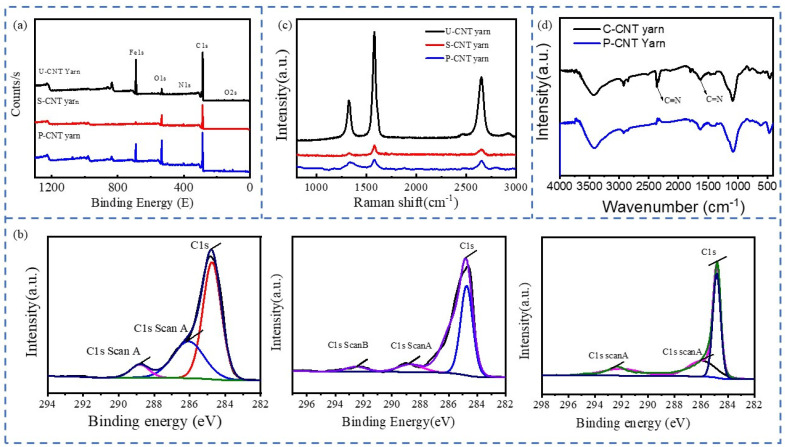
XPS plots (**a**), C1s peak fitting curves (**b**), Raman spectra (**c**) of CNT yarns in different states, and FTIR (**d**).

X-ray photoelectron spectroscopy (XPS) serves as an efficient technique for analyzing the surface composition of CNT yarns and detecting alterations in their chemical bond. The XPS spectra and C1s peak fitting curves of U-CNT yarn, S-CNT yarn, and P-CNT yarn are presented in Figure 8.

The XPS spectra of CNT yarns in various states exhibit distinct peaks of N1s (≈400 eV), F1 (≈689 eV), and O1s (≈533 eV), as depicted in Figure 8a. With the application of supercritical CO_2_ and pre-oxidation, the oxygen and nitrogen content increases while the carbon content decreases significantly. Notably, the CNT yarn and its subsequent processing do not introduce fluorine (F), suggesting that its presence may be attributed to the sample preparation process. However, post-pre-oxidation, PAN infiltrates the yarn interior, forming a ring-like structure, effectively diffusing PAN between individual CNTs for mechanical reinforcement. Figure 8b presents the deconvoluted XPS C1s spectra of UCNT yarns, SCNT yarns, and P-CNT yarns. To quantify the elemental changes, the deconvoluted XPS C1s spectra were fitted to a Gaussian-Lorentzian peak, excluding the baseline. These spectra reveal four characteristic peaks at 284.8, 286.1, 288.8, and 292.5 eV, corresponding to sp^2^ hybridized carbon (C-C), C-O, C=O, and C-N bonds, respectively. After supercritical CO_2_ treatment and low-temperature pre-oxidation treatment, the peak area of sp2 hybrid carbon (C=C) decreases. With the addition of PAN, C-N bonds appear. The relative composition of functional groups, indicated by the ratio of the area of each peak to the total area, shows that the C-C bonds of CNT yarns are significantly reduced after low-temperature pre-oxidation. This indicates that the carbon nanotubes in the yarns have been damaged. At the same time, C=O is introduced during supercritical treatment, but it reopens during the later pre-oxidation process, indicating that PAN forms effective molecular bonds between CNT molecules, improving their mechanical properties.

The Raman spectra of CNT yarns processed via various methods are presented in Figure 8c. The results indicate that U-CNT yarns exhibit obvious defects. Following supercritical treatment, PAN molecules infiltrate into the CNT yarns, leading to significantly reduced defects. Specifically, the ratio of I_D_/I_G_ decreased from 0.378 to 0.287. However, SEM images indicate that the P-CNT yarns exhibit a higher number of defects. This observation suggests that high-temperature treatment causes the internal carbon nanotubes to shrink and form internal voids, resulting in an increase in the I_D_/I_G_ ratio to 0.771.

FTIR of CNT yarns before and after pre-oxidation are shown in Figure 8d. Around 2240 cm^−1^ is the stretching vibration peak of the acrylonitrile functional group -C≡N. In the range of 1690–1500 cm^−1^, it is the double bond stretching vibration peak. In the range of 1475–1000 cm^−1^, it is the C-H stretching vibration peak. It can be seen from the infrared spectrum that, compared with the C-CNT yarn, the intensity of the stretching vibration peak at 2240 cm^−1^ of the pre-oxidized P-CNT yarn is significantly weakened, while the intensity of the stretching vibration peak at 1600 cm^−1^ in the infrared spectrum becomes stronger. This indicates that the -C≡N bond inside the fiber decreases while the -C=N bond increases. From this, we can speculate that some aromatic ring structures appear inside the pre-oxidized carbon nanotube fiber.

## 3. Material and Methods

### 3.1. Materials

Polyacrylonitrile (PAN) powder, exhibiting a molecular weight of 1.3 × 10^6^, was acquired from Sinopec Shanghai Petrochemical Company Limited. SCNC-F400, the nascent CNT yarn, was prepared by the array spinning method with a diameter of about 210 μm, kindly provided by Suzhou Jie Di Nano Technology Co., Ltd., Suzhou, China. Dimethyl sulfoxide (DMSO) was purchased from Yonghua Chemical Technology Co., Ltd., Suzhou, China. High-purity carbon dioxide (CO_2_) was supplied by Shanghai Chenggong Gas Co., Ltd., Shanghai, China. Analytical-grade acetone was procured from Shanghai Lingfeng Chemical Reagent Co., Ltd., Shanghai, China. Methanol of 99.5% purity originated from McLean Reagent Company.

### 3.2. Sample Preparation and Methods

**Figure 9:**
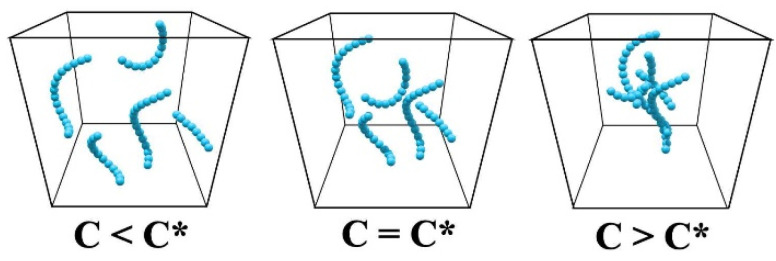
Schematic diagram of critical shear rate viscosity.

The critical overlap viscosity (C*) represents the minimal shear stress or rate necessary for achieving the critical concentration at which polymer chains begin to overlap. At concentrations beneath C*, the molecular chains remain disjointed and non-overlapping, yielding a lower viscosity state. Conversely, exceeding C* prompts chain overlap, precipitating a marked viscosity escalation, as depicted in Figure 9. This parameter is crucial for studying the molecular structure and rheological properties of polymers.

#### 3.2.1. Solution Preparation and Infiltration Treatment:

Preparation of the samples entailed an initial drying phase for moisture-sensitive polyacrylonitrile (PAN) powder at 120 °C over a 48 h period. Subsequently, 100 g of the dried PAN powder was solubilized in 100 mL of dimethyl sulfoxide (DMSO), followed by heating to 70 °C and vigorous stirring for two hours to attain a solution with a critical overlap viscosity. This primary solution was further diluted to yield concentrations equivalent to C*, half of C* (1/2 C*), one-third of C* (1/3 C*), and one-quarter of C* (1/4 C*), respectively. The CNT yarn was then submerged in each of these tailored PAN solutions for specified durations. Post-infiltration, the yarn was designated as the coated CNT yarn (C-CNT yarn).

#### 3.2.2. Supercritical CO_2_ Fluid Treatment:

**Figure 10:**
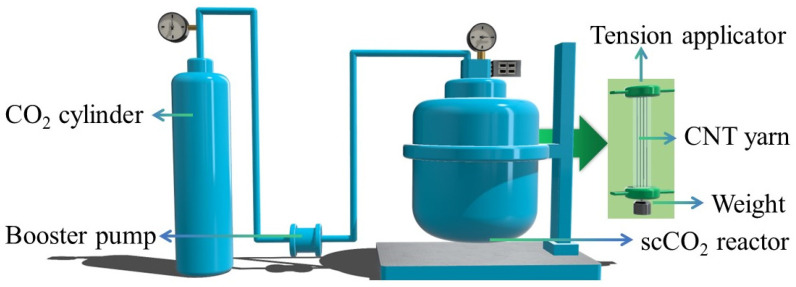
Schematic of the supercritical CO_2_ treatment setup for CNT yarn.

To treat the C-CNT yarn with supercritical CO_2_, position it within a specialized fluid reactor and affix both ends using tension clamps, applying a force range of 1.23 to 5.46 cN. Conduct repetitive cycles of CO_2_ charging and discharging under elevated pressure to purge residual air. Thereafter, elevate the reactor temperature to sustain CO_2_ in its supercritical state within the 100 °C to 200 °C temperature bracket. Manipulate the CO_2_ flow rate to attain and maintain an internal pressure of 8–12 MPa, conducive to the required reaction. Re-infiltrate the yarn with polyacrylonitrile solution and repeat the supercritical CO_2_ treatment protocol. Evaluate the structural integrity and mechanical performance of the supercritical CO_2_-modified CNT yarn (S-CNT yarn) under various tensions, temperatures, and pressures. A schematic of the supercritical CO_2_ treatment setup is depicted in Figure 10.

#### 3.2.3. Pre-Oxidation Process:

Following the supercritical carbon dioxide (sc-CO_2_) treatment, the subsequent step involves the pre-oxidation of the S-CNT yarns. This process is executed within a temperature span of 180 °C to 280 °C and within a tension span of 1.23 cN to 8.58 cN. To systematically investigate the optimal conditions, orthogonal experiments were meticulously designed with three temperature points (5, 7, 10) and distinct residence times of 1, 1.5, and 2 h at each temperature point. The pre-oxidized CNT yarn is referred to as P-CNT yarn.

### 3.3. Characterization

The microstructures of yarns were characterized using a Field Emission Scanning Electron Microscope (FE-SEM: SU8010, Hitachi Limited, Tokyo, Japan). Mechanical performance assessments of these yarns were carried out via an Instron Tensile Tester (INSTRON/5969), configured with a 2 cm gauge length and a testing velocity of 5 mm/min. Elemental compositions throughout the processing stages were analyzed by X-ray photoelectron spectroscopy (XPS: Escalab 250Xi). Additionally, a Laser Raman Spectrometer (Raman, in-Via Reflex) was utilized to assess the status of yarn defects at various processing stages.

## 4. Conclusions

A novel approach for addressing the internal micro-slippage issue in CNT yarns is introduced, leveraging the distinctive pre-oxidation and carbonization characteristics of polyacrylonitrile (PAN). Orthogonal experiments were employed to ascertain the optimal parameters for both supercritical carbon dioxide treatment and the pre-oxidation process. Under the action of supercritical CO_2_ fluid, PAN molecules penetrate into the interior of CNT yarns, interpenetrating the carbon nanotubes to fill internal gaps. Under optimal conditions, the strength of the S-CNT yarn increased by 110.10%, and the modulus increased by 100.73% compared to the U-CNT yarn.

The strength of the P-CNT yarn increased by 96.93%, and the modulus increased by 298.8% compared to the U-CNT yarn. The P-CNT yarn formed a distinct shell structure inside the CNT yarn, primarily due to the contraction of PAN molecules during the cyclization process, causing the PAN molecules to rearrange and aggregate on the surface of the CNT yarn, forming a dense shell structure. Supercritical carbon dioxide was employed to bring the PAN molecules into the gaps of the CNT yarn under the appropriate concentration. After pre-oxidation treatment, PAN cyclizes to form a dense surface layer, creating a good linking effect between the CNTs, thereby greatly improving the mechanical properties without affecting their structural components.

The limitation of this research lies in the fact that, due to the substantial workload, the interaction force between PAN molecules and CNTs has not been intensively investigated. In the future, this field can proceed in this direction. It is anticipated that in the foreseeable future, carbon nanotube fibers will undoubtedly significantly enhance their mechanical properties and become superfibers.

## Figures and Tables

**Table 1:** Orthogonal experimental table for CNT yarns.

Levels	Pressure (MPa) A	Temperature (°C) B	Tension (cN) C
1	8	100	1.23
2	10	150	4.06
3	12	200	8.40

**Table 2:** Orthogonal experimental results on the strength and modulus of CNT yarns treated with supercritical CO_2_.

Levels	A	B	C	Tensile Strength	Tensile Modulus
Factors	Pressure	Temperature	Tension	(MPa)	(MPa)
experiment 1	A1	B1	C1	179.07	5288.29
experiment 2	A1	B2	C2	167.33	3309.49
experiment 3	A1	B3	C3	138.00	4369.84
experiment 4	A2	B1	C2	229.14	3600.85
experiment 5	A2	B2	C3	277.50	5094.05
experiment 6	A2	B3	C1	73.94	2741.65
experiment 7	A3	B1	C3	131.82	3120.42
experiment 8	A3	B2	C1	138.31	3882.46
experiment 9	A3	B3	C2	171.47	5437.84
Mean intensity A	161.47	180.01	130.44	/	
Mean intensity B	193.53	194.38	189.31	/	
Mean intensity C	147.20	127.80	182.44	/	
Intensity range	46.33	66.58	58.87	B > C > A	
Mean modulus A	4322.54	4003.19	3970.80		
Mean modulus B	3812.18	4095.33	4116.06		
Mean modulus C	4146.91	4183.11	4194.77		
Modulus range	510.36	179.92	223.97		A > C > B

**Table 3:** EDS element table of CNT yarn before and after supercritical treatment.

Element	C (at%)	O (at%)	Si (at%)	Cl (at%)	Fe (at%)	Zr (at%)
C-CNT yarn	84.44	15.56	/	/	/	/
S-CNT yarn	82.66	15.99	0.20	0.38	0.62	0.15

**Table 4:** Orthogonal experimental results on the strength and modulus of pre-oxidized CNT yarns.

Levels	A	B	C	Tensile Strength	Tensile Modulus
Factors	Temperature Segments	Tension	Time	(MPa)	(MPa)
experiment 1	A1	B1	C1	188.53	8489.45
experiment 2	A1	B2	C2	192.35	8219.01
experiment 3	A1	B3	C3	189.82	10,661.99
experiment 4	A2	B1	C2	260.57	10,122.13
experiment 5	A2	B2	C3	254.84	12,984.42
experiment 6	A2	B3	C1	115.3	9323.62
experiment 7	A3	B1	C3	113.4	7115.28
experiment 8	A3	B2	C1	147.36	6762.83
experiment 9	A3	B3	C2	98.78	6277.97
Mean intensity A	190.23	187.50	150.40		
Mean intensity B	210.24	198.18	183.90		
Mean intensity C	119.85	134.63	186.02		
Intensity range	90.39	63.55	35.623	A > B > C	
Mean modulus A	9123.48	8575.62	8191.97		
Mean modulus B	10,810.06	9322.09	8206.37		
Mean modulus C	6718.69	8754.53	10,253.90		
Modulus range	4091.36	746.47	2061.93		A > C > B

## Data Availability

Data are contained within the article.
